# Overexpression of the potato *VQ31* enhances salt tolerance in *Arabidopsis*

**DOI:** 10.3389/fpls.2024.1347861

**Published:** 2024-04-05

**Authors:** Mingming Zhai, Zhengxiong Ao, Haoran Qu, Dongwei Guo

**Affiliations:** College of Agronomy, Northwest A&F University, Yangling, Shaanxi, China

**Keywords:** *StVQ31*, high salt stress, functional characterization, gene expression, physiology and biochemistry

## Abstract

Plant-specific VQ proteins have crucial functions in the regulation of plant growth and development, as well as in plant abiotic stress responses. Their roles have been well established in the model plant *Arabidopsis thaliana*; however, the functions of the potato VQ proteins have not been adequately investigated. The VQ protein core region contains a short FxxhVQxhTG amino acid motif sequence. In this study, the VQ31 protein from potato was cloned and functionally characterized. The complete open reading frame (ORF) size of StVQ31 is 672 bp, encoding 223 amino acids. Subcellular localization analysis revealed that StVQ31 is located in the nucleus. Transgenic *Arabidopsis* plants overexpressing *StVQ31* exhibited enhanced salt tolerance compared to wild-type (WT) plants, as evidenced by increased root length, germination rate, and chlorophyll content under salinity stress. The increased tolerance of transgenic plants was associated with increased osmotic potential (proline and soluble sugars), decreased MDA accumulation, decreased total protein content, and improved membrane integrity. These results implied that *StVQ31* overexpression enhanced the osmotic potential of the plants to maintain normal cell growth. Compared to the WT, the transgenic plants exhibited a notable increase in antioxidant enzyme activities, reducing cell membrane damage. Furthermore, the real-time fluorescence quantitative PCR analysis demonstrated that *StVQ31* regulated the expression of genes associated with the response to salt stress, including *ERD, LEA4-5, At2g38905*, and *AtNCED3*. These findings suggest that *StVQ31* significantly impacts osmotic and antioxidant cellular homeostasis, thereby enhancing salt tolerance.

## 1 Introduction

Salt stress significantly and adversely impacts plant growth and crop yields (Liu et al., 2022; Colin et al., 2023). Plants have evolved numerous strategies to cope with adverse environmental conditions, including but not limited to morphological, physiological, and biochemical adaptations. These mechanisms involve the transcriptional activation of relevant genes, regulating gene expression, and achieving adaptation to salt stress (Sui et al., 2017). Therefore, understanding the molecular mechanisms of plant salt tolerance has scientific importance and practical significance (Liang et al., 2023). To reveal these mechanisms, extensive studies have been conducted to identify and characterize numerous genes and proteins associated with salt tolerance, such as LRX3/4/5 proteins, CCCH-type zinc finger proteins, LncRNA973, *AtbZIP17*, etc (Zhang et al., 2019; Medina et al., 2020; Zhao et al., 2021; Gong et al., 2022). Among them, transcription factors and their regulatory functions have been demonstrated to have a crucial impact on plant environmental adaptation (Li et al., 2022).

VQ proteins that function as transcriptional regulators were first identified in *Arabidopsis thaliana*. In terms of protein structure, they possess a core FxxxVQxLTG sequence containing 5 highly conserved amino acids, where x is any amino acid (Shan et al., 2021). Subsequently, VQ genes were discovered in other organisms, such as *Arabidopsis*, rice, maize, grape, soybean, potato, bamboo, poplar, and tea, with a total of 34, 40, 61, 18, 74, 37, 28, 51, and 25 VQ members identified, respectively (Kim et al., 2013; Wang et al., 2014, 2015, 2015; Chu et al., 2016; Song et al., 2016; Wang et al., 2017; Guo et al., 2018). The VQ family has been extensively studied and, in detail and comprehensively, functionally characterized in *Arabidopsis*. VQ can interact with different transcription factors, such as WRKY and serine/threonine kinase, to regulate various stress responses in plants (Cheng et al., 2012). Moreover, the plant VQ proteins can regulate gene expression mediated by WRKY by interacting with WRKY transcription factors (Dong et al. and Petcher et al.). This interaction is achieved through the conserved residues V and Q of the FxxhVQxhTG motif (Dong et al., 2003; Pecher et al., 2014). AtCaMBP25 (AtVQ15) plays a role as a suppressor in the regulatory mechanisms of plant seedlings against osmotic stress (Perruc et al., 2004). The interaction between AtVQ9 and WRKY8 reduces WRKY8’s capacity to bind to DNA and mediate salt stress responses, thereby negatively regulating salt tolerance (Hu Y. et al., 2013). PeVQ28 enhances salt tolerance in bamboo via ABA-dependent signaling pathways (Wang et al., 2017). Additionally, salicylic acid and methyl jasmonate strongly upregulated the *AtVQ10* gene transcriptional levels in *Arabidopsis thaliana* (Chen et al., 2018). In maize, *ZmVQ19* and *ZmVQ54* were highly expressed under drought conditions. In rice, *OsVQ2*, *OsVQ16*, and *OsVQ20* also exhibited regulatory patterns similar to the maize *VQ* genes (Kim et al., 2013; Song et al., 2016). Overall, VQ proteins have been shown to play critical roles in many biological processes.

Plant performance under adverse growth conditions can be directly indicated by alterations in total protein content, chlorophyll, soluble sugars, and various other physiological parameters (Li et al., 2022; Hernández-Fernández et al., 2023). For example, when plants are stressed, the content of Pro increases rapidly and acts as a signal to regulate the expression of downstream stress-protective proteins and reduce stress damage to plants (Gu et al., 2019). MDA can seriously damage the protein and enzyme structure and the cell membrane system in plant cells, and changes in MDA content reflect, to a certain extent, the peroxidation degree of plant cell membranes (Chen et al., 2022). The antioxidant enzymes SOD, POD, and CAT can efficiently remove excess peroxide ions within cells when plants are exposed to salt stress, thereby reducing the negative impact of stress on cellular homeostasis (Xu et al., 2018). The antioxidant system’s capacity to scavenge reactive oxygen species and the extent of membrane lipid peroxidation and metabolic disorders in plants can be assessed by measuring H_2_O_2_ and O_2_^-^ (Huang et al., 2022).

In our previous study, we identified 37 VQ proteins in potato, which were classified into 6 subfamilies based on phylogenetic relationships. The qRT-PCR analysis revealed a significant upregulation of *StVQ31* in response to abiotic stress, particularly under high salinity stress (Zhai et al., 2022). As a continuation of our previous research, we focused in this paper on the functional characterization of the *StVQ31* gene. The differences in related soluble sugar, chlorophyll indexes, total protein, antioxidant enzyme activities (SOD, POD, CAT, MDA, Pro), and ROS (H_2_O_2_ and O_2_^-^) in transgenic plants were analyzed. Their correlations were also assessed, which will help us further identify the potential underlying molecular mechanisms. Furthermore, the expression of genes related to salt stress was quantitatively analyzed, which revealed that *StVQ31* regulated salt tolerance by promoting the expression of specific genes, such as *LEA*, and *AtNCED3*. Admittedly, salt stress in potato plants is complex (Wang et al., 2023), but our study adds some insights into how individual genes contribute to salt tolerance. In addition, it offers a foundation for screening candidate genes that control potato salt tolerance.

## 2 Materials and methods

### 2.1 Plant materials

The plant material used in this study was the potato cultivar Désirée provided by our laboratory. The tubers were planted in a climate chamber, in a vermiculite substrate mixed with soil, in a 1:1 ratio, v/v, for 4 weeks at a temperature of 23°C ± 1°C, 16h/8h(day/night). The seeds of WT *Arabidopsis thaliana* (Columbia (Col-0) ecotype) and 3 T3 transgenic lines (L1-L3) were vernalized for 3 days in 1/2MS medium, transferred to an artificial climate chamber at 24°C and a relative humidity of 80% for ten days, and then transplanted to a vermiculite medium with small square pots (7x7cm). Next, plants were grown for 3 weeks (Kim and Hwang, 2015). Samples were taken before and after salt stress treatment and the collected fresh leaves were quickly placed in −80°C for storage (Zhang et al., 2003). At the same time, chlorophyll content, soluble sugar, SOD, MDA, and other physiological indexes and enzyme activities were measured before and after salt stress treatment.

### 2.2 Generation of overexpression vectors and transgenic *Arabidopsis* plants

**Figure 1:**
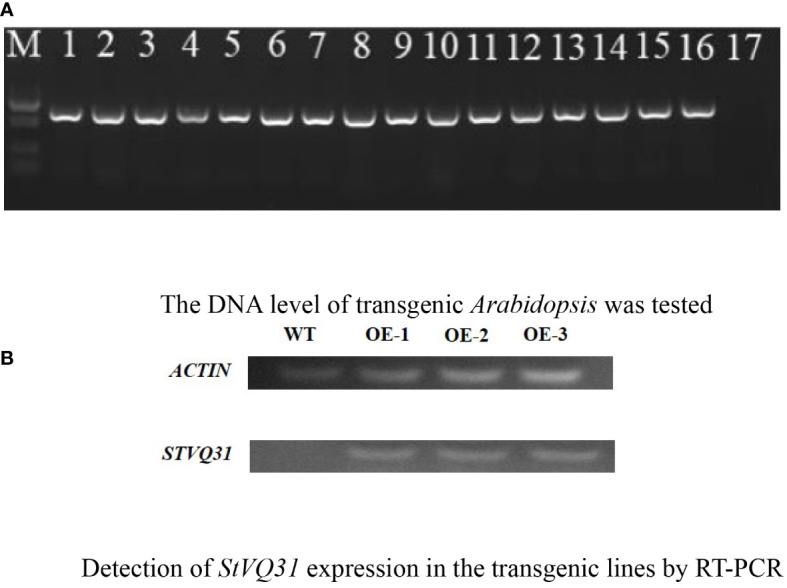
**(A)** The DNA level of transgenic *Arabidopsis* was tested; **(B)** Detection of *StVQ31* expression in the transgenic lines by RT-PCR. A:1-16:transgenic *Arabidopsis*;17: Negative control; M:DL5000 Marker B: Lane WT: wild-type; lanes *StVQ31*, transgenic plants.

The *StVQ31* gene was amplified through PCR using primers specific to the gene (**Table 1**). The CDS of *StVQ31* was ligated to the pCAMBIA1302 overexpression vector within the *KpnI* and *BstbI* restriction sites. The recombinant plasmid was verified by sequencing and was then cloned in *E. coli* DH5α. Subsequently, it was introduced into *Agrobacterium* GV3101, which was for the genetic transformation of *Arabidopsis* plants by the floral-dip method (Zhao et al., 2018). When *Arabidopsis* seeds matured, the T0 generation seeds were harvested. The T0-generation seeds were then sowed on 1/2 MS medium containing 50μg/ml kanamycin, and 16 lines were identified through PCR verification (**Figure 1A**). The above steps were repeated until T3 transgenic overexpression lines were obtained.

**Table 1:** Primers used in this research.

Primer	Primer sequence (5′-3′)	Description
StVQ31-F	TACCTCTTCTCTCTTCACTTTT	PCR
StVQ31-R	GATTTTTCCCATTTTACCCCTC	PCR
StVQ31-1300F	AGAACACGGGGGACGAGCTCGGTACC TACCTCTTCTCTCTTCACTTTT	PCR
StVQ31-1300R	CCATCATGGTCTTTGTAGTCTTCGAA GATTTTTCCCATTTTACCCCTC	PCR
HPT-F	GGTCGCGGAGGCTATGGATGC	PCR
HPT-R	GCTTCTGCGGGCGATTTGTGT	PCR
Actin-F	TCCCTCAGCACATTCCAGCAGAT	qPCR, RT-PCR
Actin-R	AACGATTCCTGGACCTGCCTCATC	qPCR, RT-PCR
LEA 4-5-F	GGAAAAGGCGGAGAAGATGA	qPCR
LEA 4-5-R	TTGTGCTGACGCGTTTCTCT	qPCR
ERD-F	GGTTGTGCGGCAGGTTATTC	qPCR
ERD-R	ATCTGCAACTTTCCCGCTGA	qPCR
At2g38905-F	TTCCTTCGATATGGTTGTGG	qPCR
At2g38905-R	GTCATCATCCGACAAGAACG	qPCR
AtNCED3-F	ATGGCTTCTTCACGGCACGG	qPCR
AtNCED3-R	TTCCTTTGCCCTCGGACG	qPCR
StVQ31-F	TGGAGCAATGGGGTTTTCGT	RT-PCR
StVQ31-R	ACAACCTCATTCCCTTCGCC	RT-PCR
StVQ31-F	ATCTCGAGCTCAAGCTTCGAAATGGCGTCTTCTGATAAT	Subcellular localization primer
StVQ31-R	CCGTCGACTGCAGAATTCGAACATTCCTGATTCAAGGGT	Subcellular localization primer

### 2.3 Prediction of StVQ31 physicochemical properties

We obtained the properties of StVQ31 from the ExPASy website, including its physical and chemical characteristics (http://web.expasy.org/protparam/) (Pedrini et al., 2015). Then, the secondary structure of StVQ31 was predicted using the PHD software (http://www.predictProtein.org/) (Sajid et al., 2018). In addition, the conserved motifs of StVQ31 were determined by MEME (version 4.12.0) (Bailey et al., 2009).

### 2.4 StVQ31 subcellular localization

The CDS region of *StVQ31* (after removal of the stop codon) was ligated to the pCAMBIA1302 vector carrying a green fluorescent protein sequence to produce pCAMBIA1302-StVQ31: GFP. The recombinant plasmid was transferred to *Agrobacterium* and then infiltrated into tobacco leaves, with the nuclear maker AtWRKY25-mCherry used as a control. All transformed tobacco plants grew in the dark at 22°C for 16 hours and then returned to normal conditions. The GFP and RFP fluorescence signals were observed by IX83-FV1200 confocal laser-scanning microscopy two days after tobacco inoculation.

### 2.5 Evaluation of seed germination and root length in the presence of salt stress

Seeds from the WT and transgenic plants were cultivated in a growth medium called 1/2 MS, supplemented with 150 mM NaCl. The seeds were incubated at 4°C for 3 days, then transferred to 22°C, with 16 hours of light/8 hours of darkness, and incubated for 10 days. Radicle appearance from the seed coat was used as the criterion for seed germination (Stacey et al., 2016). Meanwhile, seeds of WT and transgenic plants (homozygous lines) were placed in 1/2MS medium for the subsequent experiments. After 7 days, seedlings with uniform appearance and growth were selected, transplanted into 1/2 MS medium containing 150mM NaCl, and grown vertically for 7 days. Subsequently, the changes in root length were recorded. In addition, the salt tolerance of 4-week-old *Arabidopsis thaliana* transgenic plants was evaluated by irrigation with 150 mM NaCl solution every 2 days for 10 days. The phenotypes of the plants were photographed after 7 days of salt treatment.

### 2.6 Determination of relevant physiological indicators

Ten physiological indexes in WT and transgenic plants were measured before and after the application of salt treatment (150 mM NaCl). Approximately 0.5 g of leaf tissue, avoiding the midvein of the leaves, was sampled and quickly placed in liquid nitrogen. The samples were fully ground and 9 folds volumes of pH 7.4 PBS buffer were added. After centrifugation at 12,000 ×*g* at 4°C for 30 min, the supernatant was taken, and the protein concentration was determined using the BCA Protein Assay Kit, following the manufacturer’s instructions (Stanley et al., 2020). Chlorophyll was determined based on the protocol by Guo et al., with slight modifications (Guo et al., 2022). The measurement of soluble sugars was slightly modified based on the Dubois et al. (1951) method, and the content of MDA was determined by the thiobarbituric acid (TBA) method (Basit et al., 2022). The SOD activity was determined using a total SOD assay kit (the wst-8 technique; Beyotime, China) (Chen et al., 2019). Furthermore, the CAT, POD, and Pro antioxidant enzyme activities, as well as the H_2_O_2_ and O_2_^-^ content, were determined using the Solarbio Kit (Beijing, China) following the guidelines provided by the manufacturer (Ren et al., 2021).

### 2.7 Analysis of the expression of genes related to salt stress

To investigate the regulatory mechanisms of *StVQ31*, we further examined the expression changes of genes associated with salt stress. Four-week-old transgenic *Arabidopsis* were moved to MS liquid medium with 150 mM NaCl for 8 hours, while the remainder seedlings were kept in MS liquid medium as a control. Total RNA from the transgenic *Arabidopsis* leaves was extracted using the RNAprep Pure Plant Total RNA Extraction Kit (DP432), and cDNA was synthesized using the FastKing cDNA First Strand Synthesis Kit (Degenomics)(KR116) (Tiangen, Beijing, China). The template cDNA was then diluted according to experimental requirements. The NCBI primer design suite was used to design gene-specific primers (**Table 1**) with Actin as the internal reference. qPCR was performed using the ABI QuantStudio7Flex system (Applied Biosystems, USA). The reaction mixture consisted of 2 μl of cDNA, 10 μl of 2× Super Real Color PreMix SYBR (Tiangen, China), 0.6 μl of gene-specific primers, and ddH_2_O was added up to 20 μl. The reaction conditions were set to: 95 °C 15 min; 95 °C 10 s, 60 °C 32s, 72 °C 32 s, 95 °C 15 s, 60 °C 60 s, 95 °C 15 s for a total of 40 cycles. The expression levels of the evaluated genes were calculated using the 2^−ΔΔCT^ method (Livak and Schmittgen, 2001). Three biological replicates were performed for each sample.

### 2.8 Data analysis

All data were expressed as the mean ± standard errors of three replicates. Prism 7.0 was used for image rendering. The mean differences between groups were statistically analyzed by independent sample t-test, analysis of variance (ANOVA), and *post hoc* LSD test. SPSS 27.0 was used for correlation analysis.

## 3 Results

### 3.1 Cloning and bioinformatic analysis of *StVQ31*

**Figure 2:**
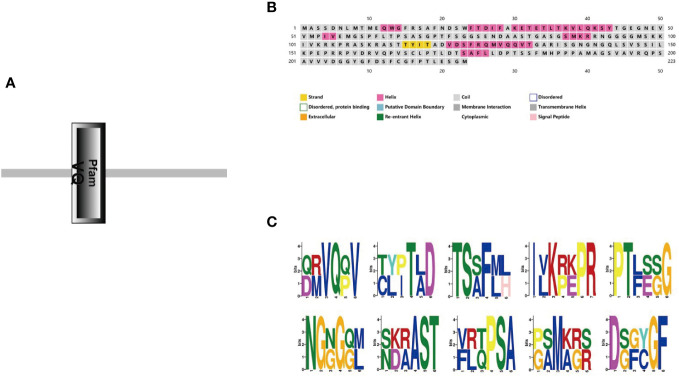
Protein structure of StVQ31 **(A)** Conservative domain of StVQ31; **(B)** Secondary structure prediction of StVQ31 Number of amino acids: 223; **(C)** Conserved motif of StVQ31.

A gene-specific amplification primer *StVQ31*-F/R was designed based on the gene sequence (**Table 1**), and total RNA was extracted from potato leaves for reverse transcription for the isolation of the complete cDNA of *StVQ31* (**Supplementary File 1A**). DNA Sequencing revealed that the *StVQ31* gene fragment amplified by PCR had an open reading frame of 672bp, encoding a protein consisting of 223 amino acids and contained a conserved VQ domain (**Supplementary File 1C**; **Figure 2A**). *StVQ31* contained only one exon. Based on the ExPASy ProtParam prediction, the theoretical MW of the encoded protein was 23.697 kDa, and the theoretical PI was 8.58. Ser (13%), Gly (10.3%), Val (7.6%), and Thr (7.6%) residues were the most abundant in the StVQ31 protein amino acid sequence. The overall mean hydrophilicity of StVQ31 was -0.295, indicating that the protein was hydrophilic. The secondary structure analysis showed that StVQ31 protein was composed by α helixes covering 19.73%, β fold covering 1.79%, and random coils covering 78.48% of the protein, respectively (**Figure 2B**). The motif analysis revealed that motif1 depicted in **Figure 2C**, served as the central conserved region of the protein and encompassed the functional domain (**Figure 2A**). In addition, the *StVQ31* gene promoter contained TGA-element cis-elements, which means it may be involved in the regulation of plant growth and development.

### 3.2 Subcellular localization of StVQ31

**Figure 3:**
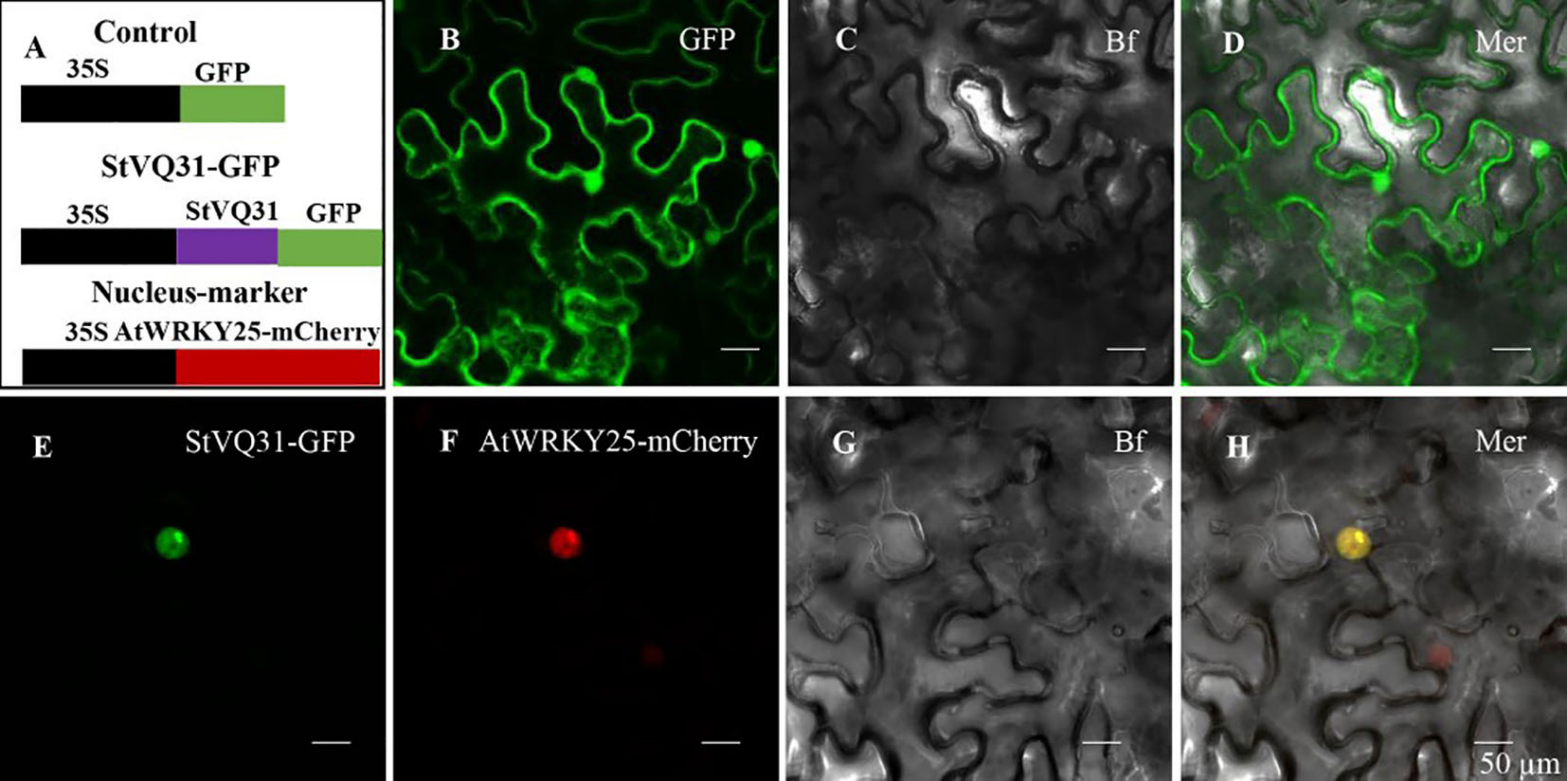
**(A) **A schematic diagram of the subcellular localization vector. **(B)** The GFP empty vector. **(E)** The recombinant 35S:StVQ31-GFP vector. **(F)** The nucleus marker AtWRKY25-mCherry. **(C, G) **Bright field. **(D, H)** Merged images. Scale bars: 50 μm.

In order to confirm the subcellular location of StVQ31, we removed the termination codon and fused StVQ31 to a GFP vector expressed by the 35s promoter of the tobacco mosaic virus (TMV). Individual cells carrying the control GFP vector exhibited detectable green fluorescence, while the cells expressing STVQ31-GFP emitted a green fluorescence signal from within the nucleus. Thus, the green fluorescence signal emitted by STVQ31-GFP could only be detected in the nucleus, and it overlapped with the red fluorescent nuclear marker AtWRKY25-mCherry (**Figure 3**), thus indicating that StVQ31 is localized in the nucleus.

### 3.3 Generation and morphological observation of transgenic plants

**Figure 4:**
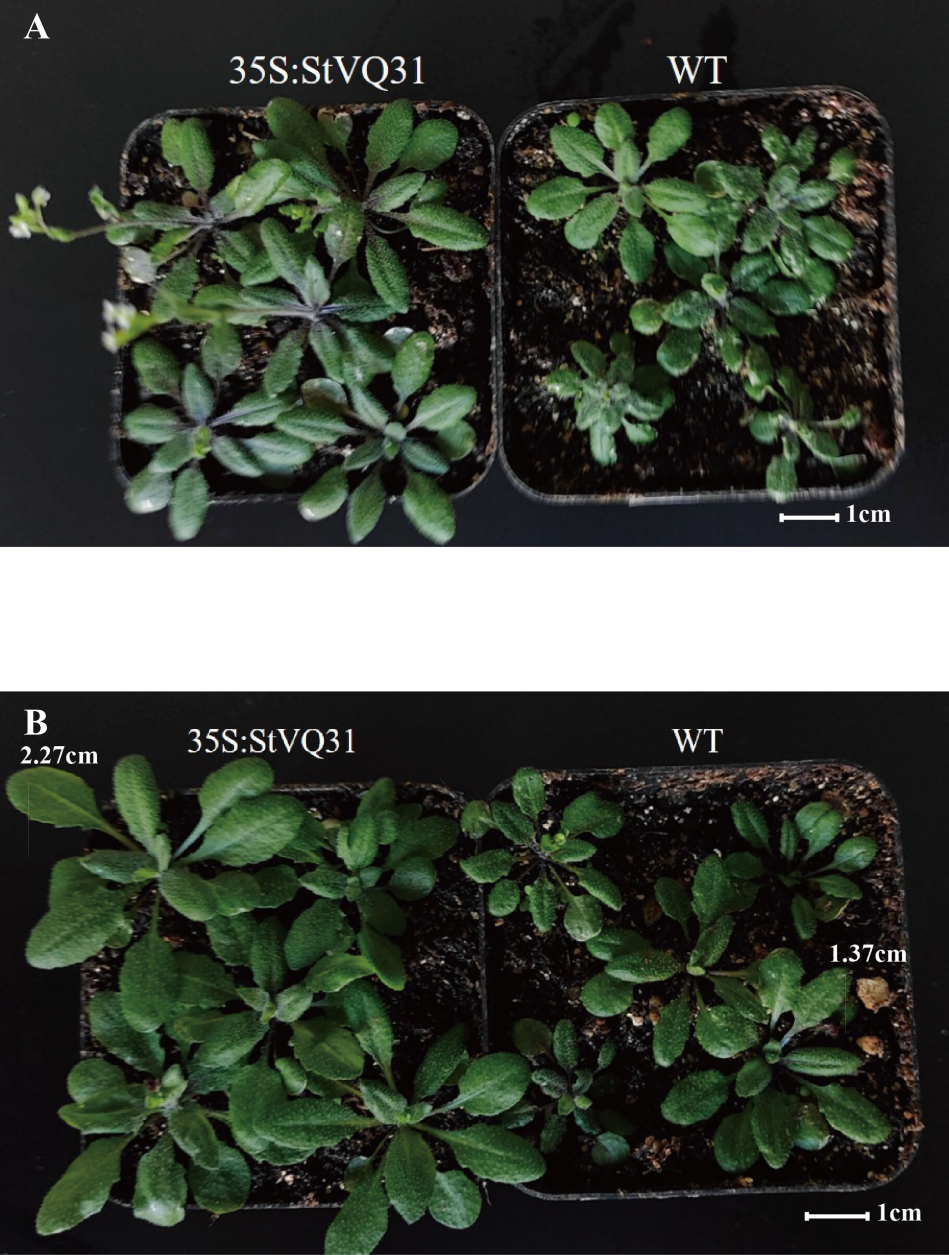
Effect of overexpression of *StVQ31* gene on flowering of *Arabidopsis thaliana* **(A)**; Effects of overexpression of *StVQ31* gene on growth and development of *Arabidopsis Thaliana* **(B)**.

The transgenic plants were identified by PCR amplification after DNA extraction. Distinct bands were amplified in the 16 transgenic lines identified. In contrast, no corresponding fragment was amplified in the WT plants (**Figure 1A**). These results were subsequently validated by RT-PCR analysis (**Figure 1B**), confirming the successful overexpression of *StVQ31* in transgenic *Arabidopsis* lines. In the early growth stages of *Arabidopsis* plants, we observed that the *StVQ31* and wild-type plants exhibited certain differences, with *StVQ31* overexpressing plants being bigger than wild-type *Arabidopsis* (**Figure 4B**). This discrepancy may be attributed to the elevated expression of *StVQ31* protein in the stem, suggesting that it is involved in the regulation of plant development. This result is consistent with the phenotypes exhibited in *AtVQ29* expressing plants (Cheng et al., 2012). Furthermore, we conducted a statistical analysis of the phenological stages and flowering dates of transgenic and wild-type *Arabidopsis thaliana* lines. We found that the flowering in transgenic plants occurred at about 30 days after germination, showing a significant increase in flowering time compared with wild-type *Arabidopsis* (**Figure 4A**). The *StVQ31* gene not only affected the flowering time of *Arabidopsis* but also affected its growth and development. This implies that it potentially has pivotal regulatory functions that control *Arabidopsis* growth. Based on this, we observed the phenotypes of transgenic and wild plants under salt stress. We found that in contrast to the wild type, the leaves of the transgenic plants exhibited a vibrant green color, while the wild type plants had yellow leaves, short plants, and were in a wilted state (**Supplementary File 2**). These results indicated that the transgenic plants exhibited an increased tolerance to salt stress.

### 3.4 Germination rate and root length

**Figure 5:**
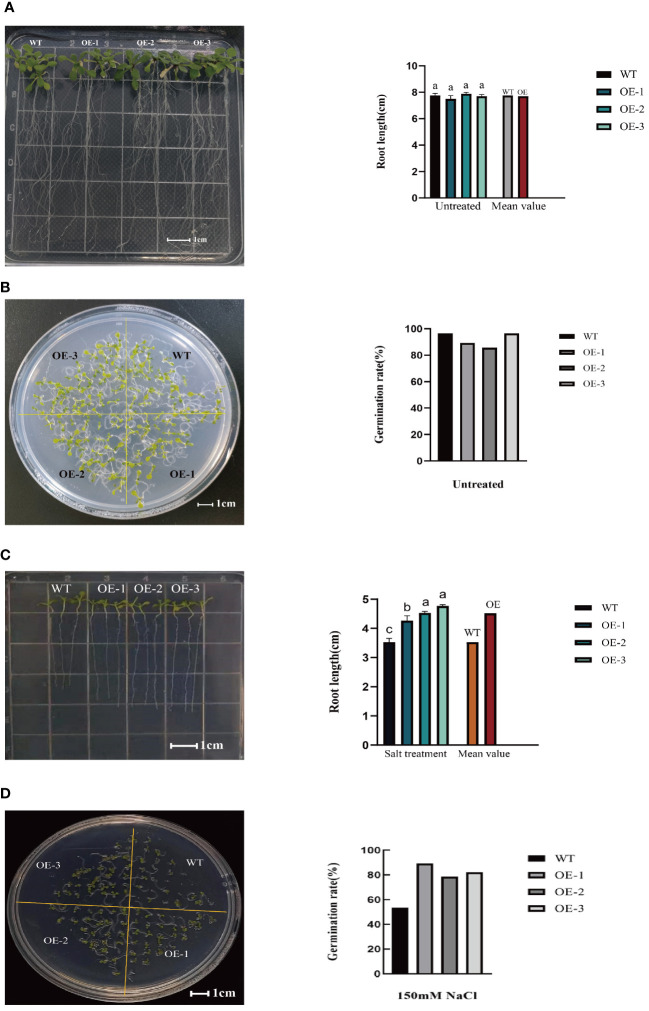
**(A)** Root length of wild *Arabidopsis* and transgenic *Arabidopsis* under 0mM NaCl; **(B)** Germination experiment of wild *Arabidopsis* and transgenic *Arabidopsis* under 0mM NaCl; **(C)** Root length of wild Arabidopsis and transgenic *Arabidopsis* after salt stress; **(D)** Germination experiment of wild *Arabidopsis* and transgenic *Arabidopsis* after salt stress. Values are means±SD (n=3), different lowercase letters indicate significant difference from the control (t-test) (P<0.05).

Seed germination serves as the foundation for plant growth and overall plant vigor. Hence, examining the impact of salt stress on seed germination is critical to assess the resistance of the plants. Under 0mM NaCl treatment, the germination rates of transgenic lines were consistent with that of WT (**Figure 5A**; **Supplementary File 3**). After salt treatment, the germination rates of the three transgenic lines evaluated were 89.29%, 78.57%, and 82.14%, respectively (**Figure 5D**; **Supplementary File 3**), increased by 66.67%, 46.67%, and 53.33%, respectively, compared to the WT (germination rate of 53.57%). At the same time, the transgenic plants had an increased root length. Specifically, compared to WT plant (3.53cm), the root length of the three transgenic lines was 4.27cm, 4.37cm, and 4.76cm, respectively (**Figure 5C**; **Supplementary File 3**), increased by20.96%, 23.80%, and 34.84%, respectively. While under 0mM NaCl treatment, the root length of transgenic lines were consistent with that of WT (**Figure 5B**; **Supplementary File 3**).These findings suggest that *StVQ31*overexpression reduced salinity-mediated inhibition of seed germination, conferring a stronger capacity to the seedlings to reduce salt stress damage and maintain longer root length.

### 3.5 Effects of *StVQ31* overexpression on the antioxidant metabolism under salt stress

**Figure 6:**
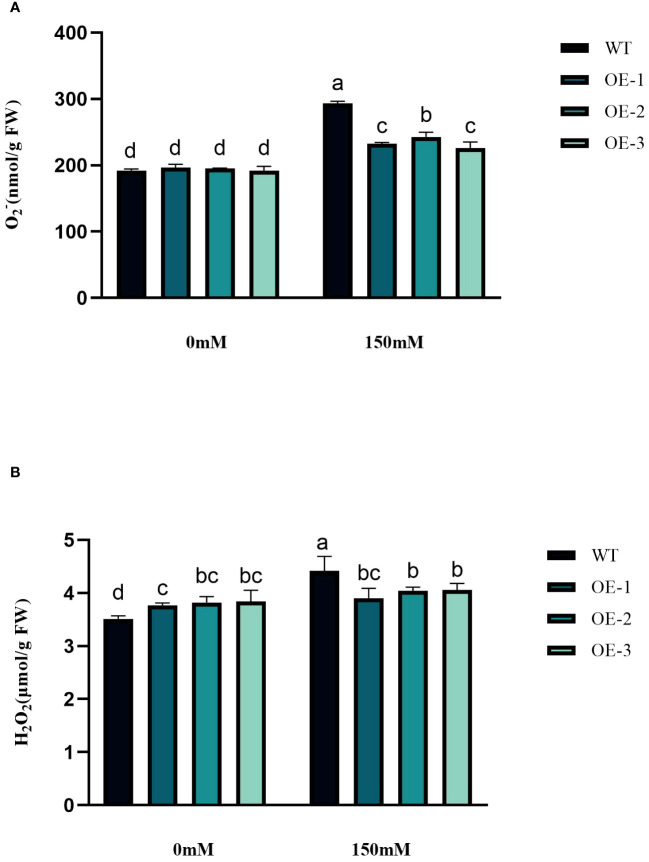
Physiological comparisons of control and *StVQ31* overexpressing *Arabidopsis* plants. **(A)** O_2_^-^; **(B)** H_2_O_2_. All values are presented as mean ± standard error of three replicates. Different letters indicate significant differences in comparisons (P < 0.05).

**Figure 7:**
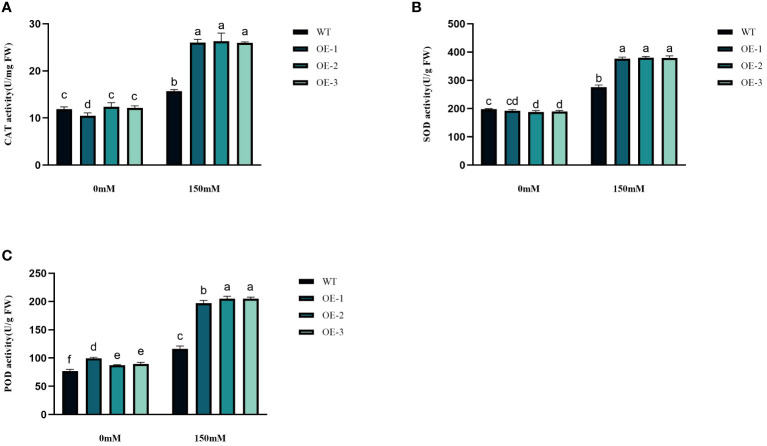
Physiological comparisons of control and *StVQ31* overexpressing *Arabidopsis* plants. **(A)** CAT activity; **(B)** SOD activity; **(C)** POD activity. All values are presented as mean ± standard error of three replicates. Different letters indicate significant differences in comparisons (P < 0.05).

Under abiotic stress conditions, considerable amounts of excessive ROS, such as H_2_O_2_ and O_2_^−^ are generated within plant cells. This leads to a rise in lipid peroxidation and subsequent cellular oxidative stress (**Figures 6A, B**). The contents of H_2_O_2_ and O_2_^−^ in *Arabidopsis* leaves were determined before and after salt treatment. The H_2_O_2_ and O_2_^−^ contents in WT under salt stress were 1.26 and 1.53 times higher than in the control conditions. The H_2_O_2_ and O_2_^−^contents increased by 3.45%~5.76% and 17.89%~24.33%, respectively, while the WT increased even more. The increase of transgenic plants was significantly lower than that of WT (**Figures 6A, B**). The WT plants, overall, did not exhibit any notable variation. Next, we measured the activities of CAT, POD, and SOD. CAT catalyzes H_2_O_2_ breakdown, while SOD and POD are responsible for the reduction of stress-induced H_2_O_2_ and O_2_^−^, respectively. Under salinity stress treatment, the enzyme activities increased in both transgenic plants and WT in different amplitudes. Compared with WT, CAT, SOD, and POD in transgenic plants were 1.66-1.68-fold, 1.37-1.38-fold, and 1.69-1.76-fold higher than WT, respectively (**Figures 7A–C**). These results are consistent with the H_2_O_2_ and O_2_^−^ contents, suggesting that transgenic plants could reduce cell oxidative damage under stress conditions by increasing ROS clearance capacity.

### 3.6 Determination of physiological indexes under salt stress

**Figure 8:**
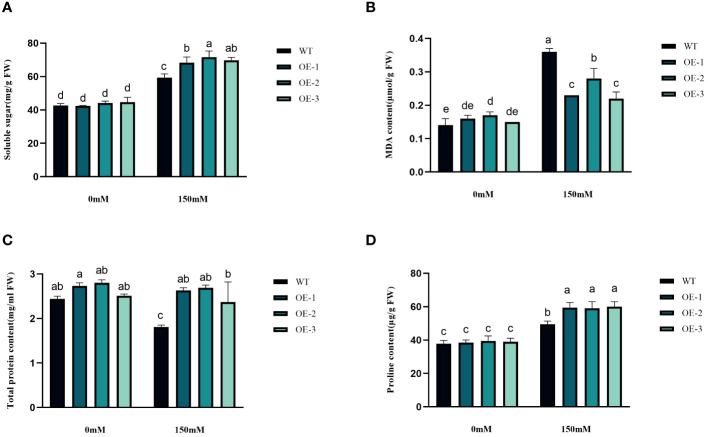
Physiological comparisons of control and *StVQ31* overexpressing *Arabidopsis* plants. **(A)** Soluble sugar; **(B)** MDA content; **(C)** Total protein; **(D)** Proline All values are presented as mean ± standard error of three replicates. Different letters indicate significant differences in comparisons (P < 0.05).

**Figure 9:**
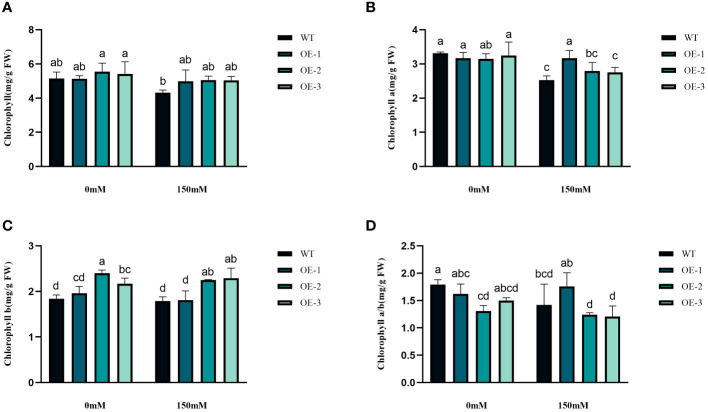
Physiological comparisons of control and *StVQ31* overexpressing *Arabidopsis* plants. **(A)** Chlorophyll; **(B)** Chlorophyll a; **(C)** Chlorophyll b; **(D)** Chlorophyll a/b. All values are presented as mean ± standard error of three replicates. Different letters indicate significant differences in comparisons (P < 0.05).

We examined the alterations in proline levels, a significant stress indicator, before and after subjecting plants to salt stress. The results found that The Pro content in WT increased 1.31-fold after salt stress, while the Pro content in transgenic plants was 1.5-1.54-fold increased (**Figure 8D**). Thus, the increase of Pro content in transgenic plants was considerably higher than that in WT, by 1.19-1.21-fold (**Figure 8D**). MDA content analysis was conducted to evaluate the oxidative damage caused by salt stress in both WT and transgenic plants. The results showed that all *StVQ31* overexpression lines produced significantly less MDA (0.216 to 0.277µmol g^−1^ FW) than WT plants (0.36µmol g−1 FW) (**Figure 8B**). These results suggest that overexpression of *StVQ31* may contribute to maintaining membrane permeability under salt stress by enhancing their antioxidant metabolism. The accumulation of soluble sugars was similar to that of proline. After being subjected to salinity stress (**Figure 8A**), the soluble sugar content in WT exhibited a substantial increase of 39.17%. In transgenic plants, soluble sugar content showed an increase ranging from 56.22% to 62.07%. The total protein content decreased in WT decreased under salt stress by 25.82% and in transgenic plants by 3.66%-5.58%. The trend was similar to that of MDA, albeit with a different amplitude of decrease (**Figure 8C**). Thus, compared with WT, the reduction was lower in transgenic plants. The chlorophyll content is directly related to the light-harvesting process in plants. It can be used as an indicator of plant photosynthesis to determine the plant’s physiological status and salt tolerance. Under salt stress, chlorophyll content decreased, affecting plant growth and development. The contents of chlorophyll a and b and the a/b ratio decreased notably under 150 mM NaCl treatment, but the total content of chlorophyll, chlorophyll a, and chlorophyll b in the transgenic plants was significantly higher than that in the WT plants (**Figures 9A–D**). These results indicate that the photosynthetic capacity of transgenic plants after salt treatment is even higher than that of WT plants.

### 3.7 Expression of salt-stress related genes

**Figure 10:**
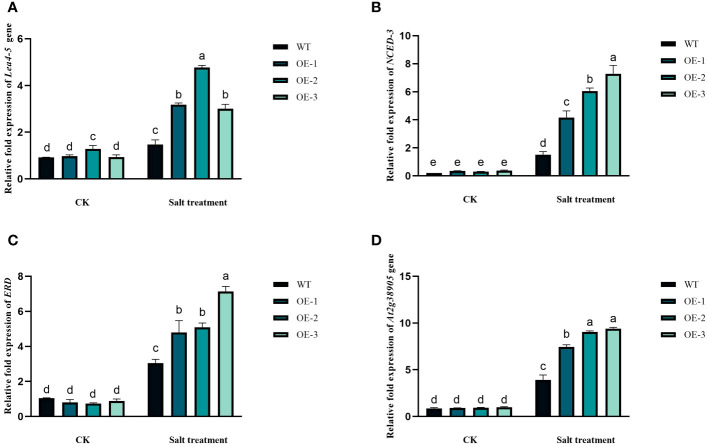
Expression analysis of abiotic stress marker genes in *StVQ31* transgenic *Arabidopsis* lines by real-time RT-PCR. The transcript levels of **(A)** *ERD*; **(B)** salt responsive gene (*At2g38905*); **(C)** *LEA 4-5*, and **(D)** *NCED-3* in WT and transgenic Arabidopsis plants were analyzed under both control and salt treatment conditions. Actin was used as housekeeping genes for normalization. Values are means ± SD (n=3), different lowercase letters indicate significant difference from the control (t-test) (P<0.05).

The involvement of the *StVQ31* gene in the adaptive response of plants to salt stress was determined by analyzing the expression of four genes that function as stress markers. Under high salinity conditions, the expression of the genes encoding *LEA 4-5, NCED-3, ERD*, and *At2g38905* was notably upregulated in transgenic plants, as shown in **Figures 10A–D**. While under non-stress conditions, their expression in transgenic plants and wild-type plants remained unaltered. Taken together, these findings suggest that the enhanced salt tolerance in plants that overexpress *StVQ31* genes is also associated with increased expression levels of *LEA 4-5, NCED-3, ERD*, and *At2g38905genes.*

### 3.8 Correlation analysis

**Figure 11:**
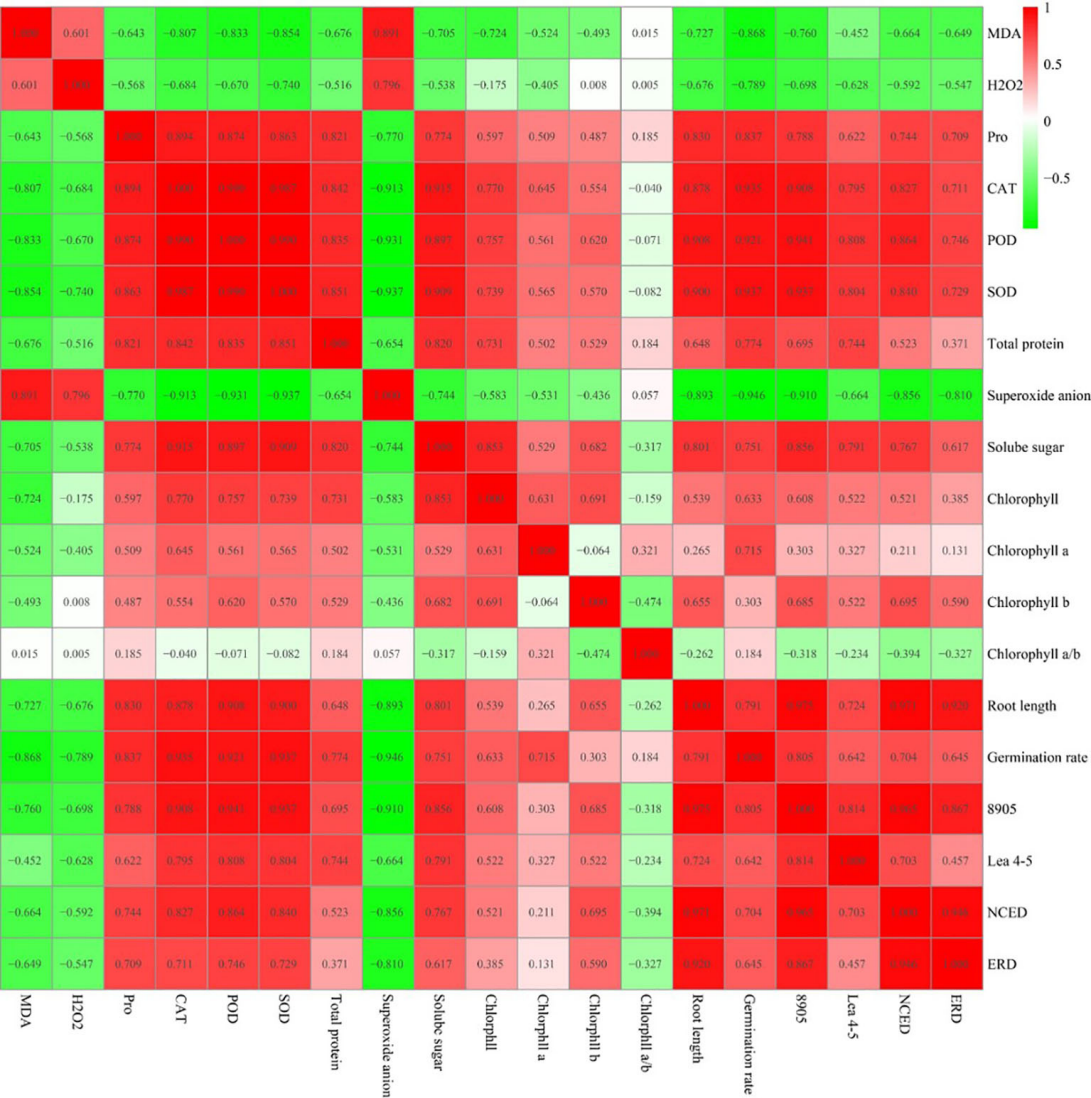
Heat map of correlation between physiological indexes after salt stress.

Based on the results, 61.99% of the measured indicators were correlated, of which 47.95% were strongly correlated and 39.77% were weakly correlated (**Figure 11**). Root length was found to have a positive correlation with SOD and POD activities (P<0.01), while the levels of superoxide anion and MDA were significantly negatively correlated. This suggests that reactive oxygen species play a role in regulating root growth. Similarly, the germination rate showed a positive correlation with SOD and CAT, and a negative correlation with superoxide anion and MDA levels. These findings indicate that superoxide anion and MDA have varying degrees of negative regulatory effects on both germination rate and root length. Moreover, total protein, SOD, POD, CAT, and Pro showed positive correlations with other indexes, whereas MDA and H2O2 were predominantly negatively correlated with other indexes. Specifically, CAT was highly correlated with SOD and POD. Root length was highly correlated with the expression of At2g38905 genes and NCED-3. Moreover, certain indexes, such as H2O2 and Chlorohyll b, were not correlated. The findings suggest a possible correlation between the indexes, which could collectively impact the salt tolerance of the StVQ31 overexpressing plants.

## 4 Discussion

VQ protein is a plant-specific protein (Wang et al., 2010). Growing evidence indicates that *VQ* regulates plant response to various abiotic stresses, including salinity, drought, cold and high temperature (Kim et al., 2013; Song et al., 2016; Wang et al., 2017; Chen et al., 2018). In *Arabidopsis thaliana*, *AtVQ9* and *AtVQ15* genes significantly affect its salt stress tolerance (Perruc et al., 2004; Hu et al., 2013). In our previous study, we identified 37 *VQ* genes in potato, which were classified into 6 subfamilies based on evolutionary tree relationships. Based on the phylogenetic tree, *StVQ31* is the closest to *AtVQ24* in phylogeny and is also phylogenetically close to *AtVQ15* (Zhai et al., 2022). Based on similar evolutionary functions of the same subfamily (Wang et al., 2015), *StVQ31* may have similar functions to *AtVQ15*. However, the function of the *StVQ* gene has not been previously investigated. Combined with previous studies and the literature, we selected *StVQ31*, a gene potentially involved in abiotic stress adaptation, for further functional analysis. *StVQ31* has a typical VQ conserved domain, and based on its gene structure, it contains only one exon. In addition, the *StVQ31* protein is also hydrophilic, with an average hydrophilic coefficient of -0.295. Subcellular localization revealed that StVQ31 is a nuclear-localized protein (**Figure 3**).

In this study, we cloned the *StVQ31* gene and transformed it in *Arabidopsis*. Based on the morphological analysis results, transgenic plants exhibited a relatively improved growth compared to WT under high salt conditions (**Supplementary File 2**). These observations indicated that *StVQ31* may be involved in plant salt stress adaptation and tolerance. Our findings are similar to the results of Kim et al (Kim et al., 2013). Seed germination is considered to be greatly affected by stress and is regarded as a crucial phase in the growth cycle of crops (Chen et al., 2020). A higher germination rate reflects a better growth capacity of the seedlings, which is essential for achieving a high yield during maturity (Wilson et al., 2014). Based on the germination rate experiment results, the survival rate of transgenic plants was higher, indicating that transgenic plants had greater salt tolerance. In addition, plant roots are important organs that absorb water and nutrients, and vigorous root growth directly affects the capacity of plants to adapt to environmental stress (Zhou et al., 2013). The taproot length of all transgenic strains was significantly longer than that of WT. Thus, overexpression of the *VQ31* gene could promote the root growth of *Arabidopsis thaliana* under salt stress, increase the root absorption area, and improve the salt tolerance of the plants. Therefore, we speculate that the *StVQ31* gene is involved in morphogenesis, growth, and development in *Arabidopsis thaliana*.

Plants have evolved various mechanisms to adapt to environmental changes (Yang et al., 2020). When plants are subjected to abiotic stress, large amounts of reactive oxygen species are produced. The plant antioxidant mechanisms can eliminate the damage caused by excessive ROS accumulation by regulating CAT, SOD, POD, and other antioxidant enzyme activities (Yang et al., 2017). The accumulation of osmoregulatory substances is an important indicator of plant stress tolerance. The accumulation of proline, soluble sugars, and total proteins can protect against oxidative stress by scavenging free radicals (Qiu et al., 2020). After salt stress, the increase of Pro content in transgenic plants was significantly higher (by 1.19-1.21-fold) than that in WT. Increased Pro content can account for elevated osmotic pressure, reducing the intracellular water potential and enhancing plant water conservation (Li et al., 2013). The change in soluble sugar content was similar to that of Pro, while the total protein content decreased to different degrees. It is speculated that these osmoregulatory compounds also help maintain the integrity of the cellular membranes (Kok et al., 2021; He et al., 2022). Malondialdehyde production and accumulation is also a common indicator of reactive oxygen species accumulation and oxidative stress in plant cells. Under salt stress, the MDA content of WT plants was higher than that of *StVQ31* transgenic plants, indicating that overexpression of *StVQ31* may lead to increased resistance to salt stress-induced oxidative stress (**Figure 8B**). In addition, the maintenance of chlorophyll and its reduced breakdown significantly affects the photosynthetic efficiency under stress (Ahammed et al., 2013). Based on our results, the chlorophyll content of transgenic lines was significantly higher than that of WT under high salt conditions. Therefore, overexpression of *StVQ31* in *Arabidopsis* may enhance its photosynthetic capacity by the maintenance of chlorophyll content, thus ensuring normal plant growth and development under stress. This is consistent with the phenotypic observations of the transgenic plants, which exhibited robust growth under salt stress. Enhanced antioxidant enzyme activities increased the capacity of transgenic plants to eliminate peroxy ions, reduced the peroxidation of cells’ plasma membrane, and improved the survival ability of plants under high salinity conditions. Furthermore, the H_2_O_2_ and O_2_^−^ levels in transgenic plants were remarkably lower than those of WT, this is consistent with the results of enzyme activity determination (Song et al., 2016).

Expression analysis of salt stress response genes could, in part, explain the molecular mechanism of salt tolerance derived from the *StVQ31* gene overexpression (Shkolnik et al., 2019). *AtNCED3*, a crucial gene for ABA production in *Arabidopsis thaliana*, plays a definite role in regulating stomatal closure and enhancing the plant’s salt stress tolerance through the synthesis and accumulation of ABA in the leaves (Lv et al., 2011). Based on our results, the expression of *NCED3* in transgenic plants was 2.65-4.48-fold higher than in WT. These results indicated that *StVQ31* could promote the expression of salt-stress-related genes and positively regulate ABA biosynthesis and signal transduction pathways, thereby improving plant salt tolerance. In addition, the high expression level of *AT2g38905* (salt-responsive protein family) in transgenic plants is consistent with the increase in the concentration of osmoprotectants under salinity conditions (Mishra et al., 2021), which leads to improved plant salt tolerance. The upregulation of *LEA 4-5* indicates that protein and membrane integrity preservation and the sequestration of ions are crucial in salt stress adaptation and tolerance (Huang et al., 2018). Erd-encoding proteins can protect macromolecules and membranes under stress (Kasuga et al., 2004), consistent with our findings of higher expression levels of *ERD* in transgenic plants under salt stress compared to WT plants.

A high salinity environment can lead to increased oxidative stress in plants, affecting normal growth and metabolism (Hasanuzzaman et al., 2021). The overexpression of the *StVQ31* gene may promote and enhance the activity of the antioxidant system by reducing the content of MDA, reducing the damage by oxidative stress, and reducing the osmotic stress damage caused by high salinity by increasing the content of osmoprotectant and osmoregulatory compounds. In addition, the *Arabidopsis’s* tolerance to salt stress can be improved by regulating the expression of stress-responsive genes. However, more work is needed to elucidate the *StVQ31* gene’s precise mechanism of action. Based on the findings of our study, *StVQ31* can be exploited to enhance salt tolerance in other plants via genetic engineering.

## 5 Conclusion

In this study, a *VQ* gene, *StVQ31*, was cloned from potato, and it was functionally characterized by overexpression in transgenic *Arabidopsis thaliana* plants. *StVQ31* overexpression significantly enhanced *Arabidopsis* tolerance to salt stress in terms of growth and its overall physiological status. Salt stress significantly enhanced the activity of the antioxidant enzyme system and maintained the stability of membrane lipids and reactive oxygen species homeostasis. At the same time, it also resulted in changes in the germination capacity and flowering of *Arabidopsis thaliana* seedlings under salt stress. In addition, overexpression of *StVQ31* induced the expression of genes involved in salt stress adaptation. The results showed that the *StVQ31* gene plays a critical role in plant responses to salt stress. However, due to the complexity of *StVQ31’s* function, its precise mechanism of action still needs to be further explored.

## Data availability statement

The original contributions presented in the study are included in the article/**Supplementary Material**, further inquiries can be directed to the corresponding author/s.

## Author contributions

MZ: Conceptualization, Formal analysis, Software, Visualization, Writing – original draft, Writing – review & editing. ZA: Formal analysis, Software, Writing – review & editing. HQ: Formal analysis, Software, Writing – review & editing. DG: Conceptualization, Funding acquisition, Writing – review & editing.

## Supplementary material

Supplementary file 1 Determination of total RNA in potato leaves by agarose gel electrophoresis: Agarose gel electrophoresis of *StVQ31* cDNA amplification and PLB- *StVQ31*positive identification A:M: DL2000 DNA Marker, 1-5: RNA; B: 1-2: blank control, 3-4: the PCR detection result of *StVQ31*-1300 bacterial solution, M: DL 2000 DNA Marker C: cDNA amplification of *StVQ31* gene; D: positive identification of two single colonies of PLB- *StVQ31*; M: DL2000 DNA Marker, 1-2: StVQ31.

Supplementary file 2 Morphology of wild *Arabidopsis* and transgenic *Arabidopsis* before and after salt stress (150mM NaCl).

Supplementary file 3 Root length and germination rate of wild *Arabidopsis* and transgenic *Arabidopsis* before(0 mM NaCl) and after salt stress (150mM NaCl).
