# Supplementary figures and images for: Overexpression of the potato VQ31 enhances salt tolerance in Arabidopsis
Source: Front Plant Sci. 2024 Apr 5;15:1347861. doi: 10.3389/fpls.2024.1347861 (PMC11027747; doi:10.3389/fpls.2024.1347861)

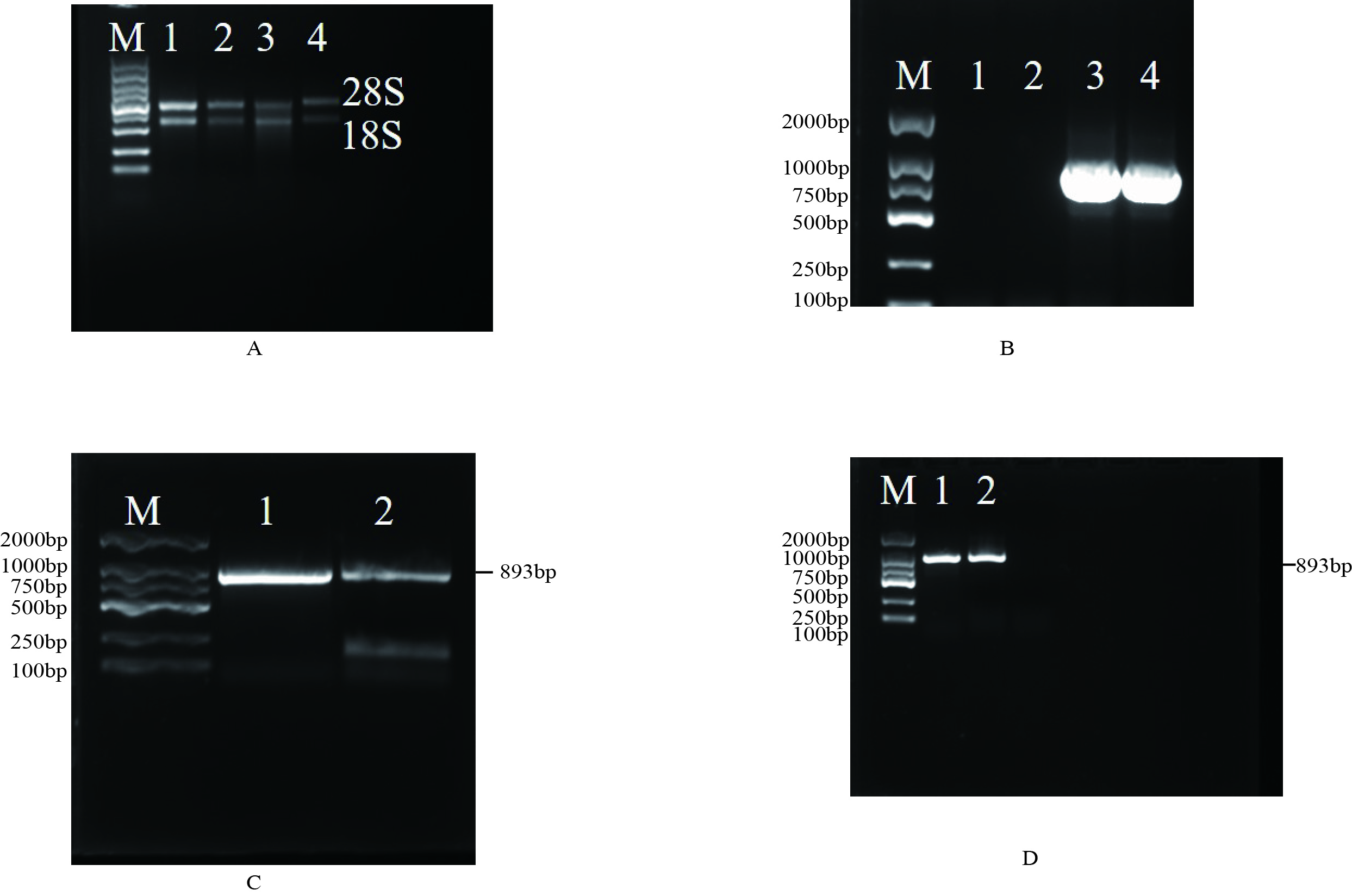

Supplement: Supplementary file 1 — Determination of total RNA in potato leaves by agarose gel electrophoresis: Agarose gel electrophoresis of StVQ31 cDNA amplification and PLB- StVQ31positive identification A:M: DL2000 DNA Marker, 1-5: RNA; B: 1-2: blank control, 3-4: the PCR detection result of StVQ31-1300 bacterial solution, M: DL 2000 DNA Marker C: cDNA amplification of StVQ31 gene; D: positive identification of two single colonies of PLB- StVQ31; M: DL2000 DNA Marker, 1-2: StVQ31. [file Image_1.jpeg]

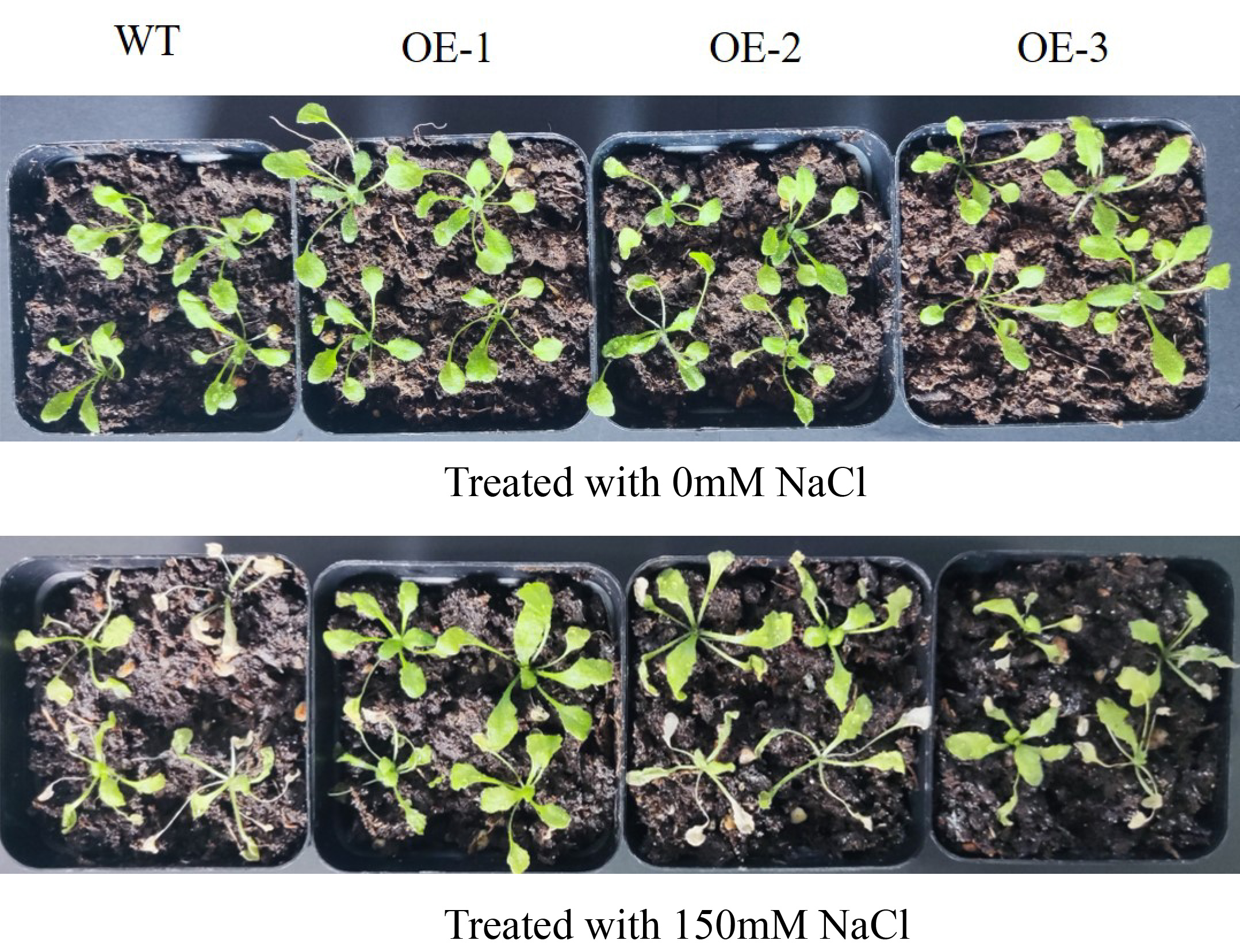

Supplement: Supplementary file 2 — Morphology of wild Arabidopsis and transgenic Arabidopsis before and after salt stress (150mM NaCl). [file Image_2.jpeg]

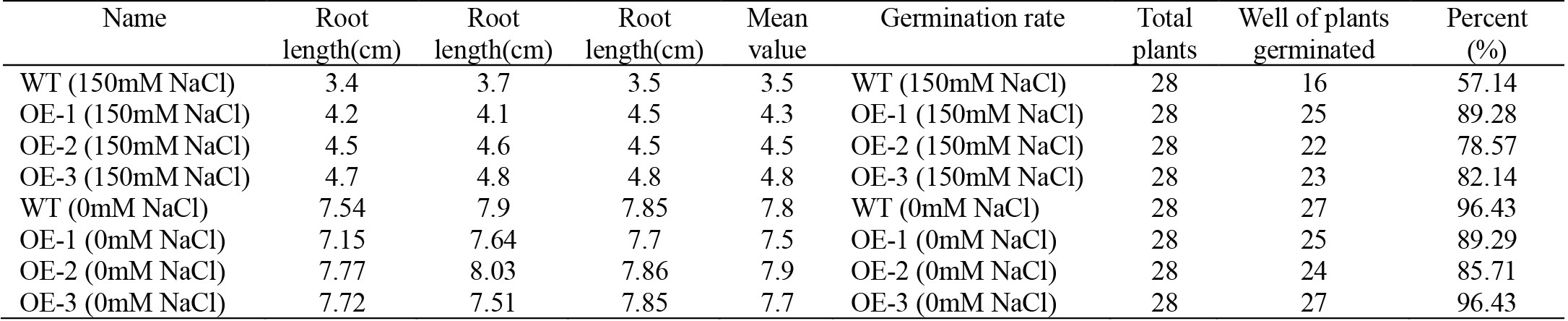

Supplement: Supplementary file 3 — Root length and germination rate of wild Arabidopsis and transgenic Arabidopsis before(0 mM NaCl) and after salt stress (150mM NaCl). [file Image_3.jpeg]
